# Bioactive compound identification without fractionation: an *Ocimum* spp. case study

**DOI:** 10.1007/s11306-025-02369-2

**Published:** 2025-11-15

**Authors:** Evelyn J. Abraham, Kelsey Custer, R. Teal Jordan, Joshua J. Kellogg

**Affiliations:** 1 Intercollege Graduate Degree Program in Plant Biology, Pennsylvania State University, University Park, PA 16802 USA; 2 Department of Veterinary and Biomedical Sciences, Pennsylvania State University, University Park, PA 16802 USA

**Keywords:** Biochemometrics, Metabolomics, Machine learning, Cytotoxicity, Ocimum, Basil, LASSO regression

## Abstract

**Introduction:**

Identifying the phytochemistry underpinning a plant’s observed therapeutic benefits is essential for understanding mechanisms of action and developing novel therapeutics. More recent efforts fusing global metabolomics and multivariate predictive modeling have improved compound discovery; however, these models rely on chemical variations between samples, which often necessitates at least one round of fractionation and may result in compound loss or degradation.

**Objectives:**

This study uses multiple whole botanical extracts to explore whether a metabolome-wide association study approach can accurately identify bioactive phytochemicals without prior fractionation.

**Methods:**

We employed 40 *Ocimum* extracts with a range of IC_50_ levels against HT-29 cells in an in vitro MTT assay and combined this data with untargeted UPLC-MS/MS metabolomics for biochemometric modeling of the potential bioactives. Multiple chemometric tools and statistical filters were employed to improve feature selection.

**Results:**

The metabolomic profiles resulted in ca. 1600 metabolite features; implementing source-based filters, followed by LASSO dimension reduction, improved the reliability of Partial Least Squares (PLS) bioactivity predictions. The resulting model highlighted four biomarkers positively correlated with activity, one of which was putatively identified as gallic acid. Gallic acid’s cytotoxicity against HT-29 cells was confirmed with the purified compound.

**Conclusion:**

This study results demonstrated that predictive modeling of botanicals using a metabolome-wide association study of extracts with no fractionation was capable of identifying biologically active compounds.

**Supplementary Information:**

The online version contains supplementary material available at 10.1007/s11306-025-02369-2.

## Introduction

Herbal products have long been regarded as valuable sources of potential bioactive compounds, and many available medicines are based on plant-derived compounds; one famous example is the anticancer drugs docetaxel and paclitaxel discovered from Pacific yew (*Taxus brevifolia*) (Vaishampayan et al., 1999). The multitude of potential medicinal compounds contained in plant tissues results in numerous therapeutic effects, including anti-inflammatory properties (Ghasemian et al., 2016), anti-microbial potential (Sumathi & Parvathi, 2010), and mental disorder treatments (Kenda et al., 2022). Plant-based treatments have formed the basis of pharmacopeia for millennia across the globe and are increasingly popular in modern cultures. The dietary supplement industry, which includes herbal products, is expecting a compounded annual growth rate of 9.1% by 2030, following a spike in 2020 as a response to the COVID-19 pandemic (Lordan, 2021).

To successfully and safely develop new pharmaceuticals or dietary supplements, the compounds responsible for the observed therapeutic actions must be characterized. Identification of these bioactive compounds allows researchers to investigate mechanisms of action, determine the optimal dosage, and generate targeted drug therapies. Additionally, dietary supplement regulations (e.g., the Dietary Supplement Health and Education Act of 1994 (DSHEA) require labels to include potency, which is often measured based on the concentration of active compounds in the formulation (Young et al., 2018). Beyond drug and supplement formulations and regulations, knowledge of compounds associated with therapeutic effects can guide plant breeding and harvesting programs, so that plant varieties are optimized to maximize medicinal benefits (Wang et al., 2020).

The traditional route to bioactive compound discovery, bioassay-guided fractionation, uses iterative chromatography stages to separate the thousands of compounds in an active plant into smaller subsets, which are subsequently analyzed to find those that retain the activity. This is repeated until a single compound with the desired biological effect remains (Mani et al., 2022). While this approach has been successful in many cases (Sabotič et al., 2024; Weller, 2012), it is well-suited for highly active compounds, but labor and time requirements hinder the rate of novel compound discovery. Additionally, at each fractionation step, there is a risk of losing low-abundance compounds with potential activity or separating metabolites that act in concert to provide activity (Kellogg et al., 2016). Further, bioassay-guided fractionation uses an excess of solvents that do not align with the current push for green chemistry (Welton, 2015).

The emerging field of biochemometrics focuses on introducing and optimizing multivariate statistical models to overcome many of these limitations. Biochemometrics combines the untargeted metabolite profiles from extracts, which often contain thousands of unknown compounds, with bioactivity information to discover the compounds associated with desired biological effects (Kellogg et al., 2016). These models are built around correlating phytochemical variations between samples (usually quantitative or semi-quantitative variations) with the observed biological activity. In most cases, Partial Least Squares – Discriminatory Analysis (PLS-DA) or PLS-Regression (PLS-R) are used as supervised approaches to identify the metabolite features that covary with changes in bioactivity. PLS-DA is successful in separating active from inactive groupings, while PLS-R highlights features correlating with shifts in the bioactivity (Abraham & Kellogg, 2021). However, most instances use at least one fractionation step to identify compounds from a single active plant. This fractionation step limits the chemical diversity in each sample, allowing for a gradation of features considered in subsequent models, improving model accuracy and compound discovery (Kellogg et al., 2016). However, recent studies suggest that stringent data filtering and preselection can improve biochemometric analyses, which may eliminate the need for an initial fractionation stage (Janairo et al., 2021; Kirpich et al., 2018; Schiffman et al., 2019). Additionally, there is potential to compare numerous botanical varieties and species simultaneously as opposed to a single plant sample.

The present study aims to investigate the possibility of a biochemometric approach to identify compounds associated with shifts in bioactivity without fractionation of the plant extract. Instead, this approach utilizes multiple botanical extracts in a single bioassay stage to simplify the compound discovery workflow for streamlined applications. We hypothesized that the inclusion of multiple global metabolomes would provide a metabolome-wide association study to model the phytochemistry, and introducing strict feature filters and LASSO preselection would reduce PLS-R model complexity to include only influential metabolites and identify those most responsible for bioactivity variations.

To test this goal, we combined the cytotoxicity of 40 *Ocimum* (basil/Tulsi) products against the HT-29 (human colorectal cancer) cell line in an MTT assay with untargeted UPLC-MS/MS data as a proof-of-concept investigation. There are many species and cultivars of *Ocimum*, some of which are commonly called Tulsi, and are associated with a range of therapeutic properties. This includes *Ocimum tenuiflorum* L. and *Ocimum gratissimum* L. which are the most common *Ocimum* species used for herbal commerce (Joshi, 2013). *Ocimum basilicum* L., or sweet basil, is primarily used as a culinary herb, but also has many reported therapeutic benefits (Dev Sharma et al., 2022). The range in reported uses, therapeutic properties, and chemical profiles of these *Ocimum* species was ideal for this investigation because it resulted in a range of biological activity levels and metabolite concentrations. We used these variations to build a PLS-R model for bioactive compound identification following strict feature prefiltering, a LASSO feature selection stage, and multiple feature importance criteria that ultimately determined phytochemical associations correlating with variations in cellular proliferation activities. Notably, we annotated one bioactive compound as gallic acid and confirmed its cytotoxicity in a subsequent assay.

## Materials and methods

### Chemicals and reagents

The solvents and chemicals in this study were obtained from VWR (Radnor, PA, U.S.A.) or Sigma Aldrich (St. Louis, MO, U.S.A.) and were of reagent of spectroscopic grade as needed.

### *Ocimum* material collection

Two sets of *Ocimum* samples were grown or procured for this study:

A. Greenhouse growth: 25 of the 40 materials used in the present study were grown in a greenhouse under controlled conditions at the Pennsylvania State University. This included 11 *O.* *basilicum*, 10* O.* *tenuiflorum*, and 5 *O.* *gratissimum* materials. Sample information is provided in Supplemental Table S1. Five of each variety were grown with a 16:8 light: dark cycle in the same greenhouse in 4 inch pots with standard potting soil. Once a plant showed its first inflorescence, but before flowering, all leaves on each plant were harvested at the base with sterile scissors and immediately flash frozen in liquid nitrogen. The five replicates were combined prior to extraction per sample, with three biological samples obtained per sample. Taxonomic designations were authenticated using seed morphology, as described in Abraham et al. (2025).
B. Consumer products: Additionally, we ordered 16 commercially- available bulk *Ocimum* herbal materials. This included 4 *O. basilicum*, 7 *O.* *tenuiflorum*, and 5 *O.* *gratissimum* materials. We obtained these materials from 7 different sources, including reputable herbal ingredient companies, organic farms, and online retailers like Etsy. All materials were stored at room temperature in the dark until further processing.

### Sample extraction

All herbal materials were ground to a fine powder prior to extraction, and all extractions were performed in triplicate. Greenhouse materials were lyophilized at the Pennsylvania State University CSL Behring Fermentation Facilities before being ground under liquid nitrogen with a mortar and pestle. Consumer products were processed with a stainless-steel coffee grinder. A 1:1 (g/mL) solid-liquid extraction was performed by combining powdered material with 80% aqueous methanol with 0.1% formic acid and shaking at 200 rpm at room temperature for 16–18 h. Following incubation, solid material was removed via vacuum filtration with 0.2 μm filter paper and liquid extract was dried to completion via rotary evaporation. Extracts were stored at room temperature until further processing. Extract yields can be found in Supplemental Table S1.

### MTT assay

Human HT-29 human colorectal cells were purchased from American Type Culture Collection (Manassas, VA) and maintained in McCoy’s 5 A medium supplemented with 10% fetal bovine serum, 100 U/ml penicillin, and 100 µg/mL streptomycin at 37 °C under humidified atmosphere with 5% carbon dioxide. Cell viability was analyzed via the 3-(4,5-dimethylthiazol-2-yl)−2,5-diphenyltetrazalium (MTT) assay. Cells were seeded in 96 well plates at a starting concentration of 10 × 10^3^. HT-29 cells were treated with 5 concentrations of each extract in media for 48 h: 0 µg/ml, 25 µg/ml, 50 µg/ml. 100 µg/ml, and 200 µg/ml. Following treatment, the HT-29 cells were washed and incubated with MTT (1 mg/ml) at 37 °C for 1.5 h. Cell viability was reported as the conversion of MTT dye to formazan precipitate measured spectrophotometrically at 540 nm. Treated cell viability was normalized to medium-treated control well. Each extract was plated at each concentration 6 times/96-well plate, and the entire process was repeated 3 times. MTT is reported as the average and standard deviation of the cell viability across the 18 replicates. IC_50_ was calculated by using a four-parameter logistic regression generated from plotting % viability (y) against extract concentration (x) to determine the concentration at which 50% of cells were inhibited. IC_50_ values were log transformed prior to additional analysis.

### Ultraperformance liquid chromatography-tandem mass spectrometry analysis

All samples were prepared at a concentration of 1 mg/mL in LC-MS grade methanol with 1 μm chlorpropamide (Stanta Cruz Biotechnology, Dallas, TX, USA) as an internal standard. Chlorpropamide has served as the internal standard due to its ready ionization in both positive and negative modes and its absence from botanical and natural product samples. For each sample, all three biological replicates were analyzed via LC-MS and included in the downstream metabolomics analysis.

Untargeted metabolomic analyses were performed on a Vanquish Duo UHPLC system connected to a Thermo Orbitrap Fusion Lumos Mass Spectrometer (Thermo Fisher Scientific, Waltham, MA). A Waters Acquity UPLC BEH C18 (1.7 μm, 2.1 × 150 mm) column was used with a flow rate of 0.1 mL/min at 55 °C. Solvent A was 0.1% formic acid (v/v) in LC-MS water and solvent B was 0.1% formic acid (v/v) in LC-MS acetonitrile. The mobile phase gradient of solvent B was as follows: 3% for 0.01 min, 45% for 10 min, 75% for 2 min, 100% for 4.5 min, and 3% for 0.2 min. A 2 uL injection was used for all samples.

Mass spectrometry was conducted using an electrospray ionization source with a positive ion spray voltage of 3500 V, sheath gas pressure of 25 Arb, auxiliary gas pressure of 5 Arb, ion transfer temperature of 275 °C, and vaporizer temperature of 75 °C. MS^1^ data was acquired with an Orbitrap resolution of 120,000, scan range of 100–1000 Da, and RF lens of 50% in the profile mode. MS^2^ data was collected in a data-dependent manner using an intensity threshold of 2.5e4 and 30-second dynamic exclusion. Raw spectral data was deposited in the MASSive database (ID: MSV000094012, 10.25345/C5V980317).

### Data processing and preparation

The UPLC-MS/MS data were analyzed and processed using MZmine3.1 software (Schmid et al., 2023). Peaks were detected with a noise level of 5.5E5 counts, minimum peak duration of 0.25 min, and 25% tolerance for *m/z* intensity variation. The ADAP algorithm was used to build chromatograms with the following parameters: minimum group size = 5, group intensity threshold = 5.5E5, minimum highest intensity = 5.5E5, and scan-to-scan accuracy = 0.05 Da or 10.0 ppm. Chromatograms were resolved using the ADAP intensity window chromatogram resolution feature with a signal/noise threshold of 7, minimum feature height of 80, coefficient/area threshold of 110, peak duration range of 0.00–0.10 min, and RT wavelet range of 0.00 to 0.10 min. Isotopes were filtered before integrating all features with the join aligner algorithm with the following parameters: *m/z* tolerance = 0.05 Da or 10.00 ppm, weight for *m/z* = 50, RT tolerance = 0.25 min, weight for RT = 50, and mobility weight = 1.00.

Prior to multivariate modeling, we performed several data preprocessing steps. First, any features with zero variance were removed and zeros were replaced with a small number (0.001 We next evaluated several data transformation techniques and chose to Hellinger transform and auto-scale metabolite peak area to normalize the dataset. Other we also tested log transformations and Pareto-scaling, and found that the Hellinger transformation and auto-scaling combination resulted in the most normal data based on histograms and Shapiro-Wilk test W-values and p-values (data not shown). These steps are implemented to reduce heteroscedastic noise and reduce the impact of extreme peak areas on subsequent modeling steps.

### Principal component analysis

Principal Component Analysis (PCA) was conducted to visualize potential patterns in the untargeted metabolite data related to shifts in IC_50_. The PCA was performed with the Hellinger transformed and auto-scaled dataset prior to the data filtration steps discussed in Sect. 2.8.

### Partial least squares-regression analysis with data filtration and preselection

Partial least squares – regression analysis (PLS-R) was performed using the *mdatools* package in R. Feature peak area was Hellinger transformed before the dataset was split into 80:20 training and test sets that were balanced to ensure an equal distribution of IC_50_ levels were represented in each set. The training data was auto-scaled, and the test data was auto-scaled based on features’ means and standard deviation in the training set. The PLS-R model was constructed using Leave One Out (LOO) cross validation for internal validation using the training set, and the test set was predicted based on the optimal number of components selected through cross validation. Root mean square error was calculated to compare model accuracy in internal cross validation and test set predictions.

Following an initial PLS-R model using all features in the original dataset, a blank filter was applied to remove all features without a peak area five-fold higher than the average of the blanks in at least 8 samples. An eight-sample threshold was chosen to represent at least half of the samples in the smaller material source (consumer products) group. An additional sample-source group filter was applied to remove all features not present in all 40 samples or not present in at least half of either group so the constructed model would be less skewed by on the large observed variation between the consumer products and greenhouse grown materials (Fig. 2C).

For further feature preselection, we implemented a Least Absolute Shrinkage and Selection Operator (LASSO) model. LASSO models use a regularization parameter to reduce the coefficients of uninformative variables to zero, resulting in a simpler model with much fewer variables. We performed LASSO with the *glmnet* function in R using LOO cross validation to select the lambda with the smallest mean square error. All variables not removed from the model were selected for a subsequent PLS-R model.

To identify key features associated with shifts in bioactivity, we used a Variable Importance in Projection (VIP) and Selectivity Ratio (SR) plot. Both plots were created using functions in the *mdatools* package based on the number of components selected through cross validation in the final PLS-R model. The peak area was compared between the top 5 most and least active materials to determine if each key feature was associated with increased or decreased bioactivity.

### Key feature annotation

To annotate key features, we used the Global Natural Products Social Molecular Networking (GNPS) website for molecular networking and library matching. We screened potential library matches based on key feature *m/z*, followed by manual comparison of library and experimental spectra. From this evaluation, only one potential annotation was discovered – compound 4 was putatively identified as gallic acid. We created a mirror plot in RStudio to directly compare the library and experimental spectra.

## Results

A hindrance to botanical bioactive compound discovery is the time and labor constraints involved in bioassay guided fractionation. Even for biochemometric studies, fractionation is usually included to distribute the phytochemistry and provide variation for the modeling to correlate against bioactivity. We aimed to determine if using multiple herbal samples with a range of biological activity combined with chemometric tools can reduce the need for fractionation stages and improve metabolomics data mining. To this end, we analyzed the cytotoxic effects of 40 *Ocimum* spp. materials. *Ocimum* is an ideal genus for such an endeavor because it contains multiple species, each with several cultivars, that have reported variations in biological activities, chemical constituents, and commercial uses (Abraham et al., 2025; Zahran et al., 2020). *O. basilicum*, which is a popular culinary herb, is common in herbal commerce, in addition to many species of “Holy Basil” or Tulsi. *Ocimum* products, including teas, tinctures, and dried herb, are consistently reported to have strong antioxidant, antimicrobial, and stress relieving properties (Dev Sharma et al., 2022; Saxena et al., 2012; Yamani et al., 2016). These activities are often linked to *Ocimum*’s high levels of flavonoids, tannins, and phenolic acids (Backiam et al., 2023). Additionally, *Ocimum* contains highly aromatic essential oil compounds, including camphor, eugenol, chavicol, eucalyptol, and many others (Abraham et al., 2023; Pandey et al., 2014; Yamani et al., 2016).

However, there are varied reports about *Ocimum*’s cytotoxic effects. As a food and herbal product, *Ocimum* is considered safe to consume, but some studies report cytotoxic effects against many human cancer cell lines. Harsha et al. (2020) reported an IC_50_ range of 30–50 µg/ml of *O. tenuiflorum* extracts against the leukemic cell lines K562 in an MTT assay (Harsha et al., 2020), and Lam et al. (2017) determined that *O. tenuiflorum* has an IC_50_ less than 100 µg/mL against MCF-7 and MDA-MB-231 breast cancer lines (Lam et al. 2017). There are also reports that *O. gratissimum* inhibits breast cancer cell proliferation and pomolic acid isolated from *O. gratissimum* is as effective at inhibiting MCF-7 cells as a reference drug (Nangia-Makker et al., 2007; Nganteng et al., 2022). Other species of *Ocimum*, like *O. canum*, have cytotoxic effects against MCF-7 breast cancer cells (IC_50_ = 60 µg/ml) (Tamil Selvi et al., 2015). Interestingly, Dolghi et al. (2021) reported that at concentrations lower than 75 µg/ml *O. basilicum* extracts have a stimulatory effect on HT-29 human colorectal cancer cell growth (Dolghi et al., 2021).

**Fig. 1:**
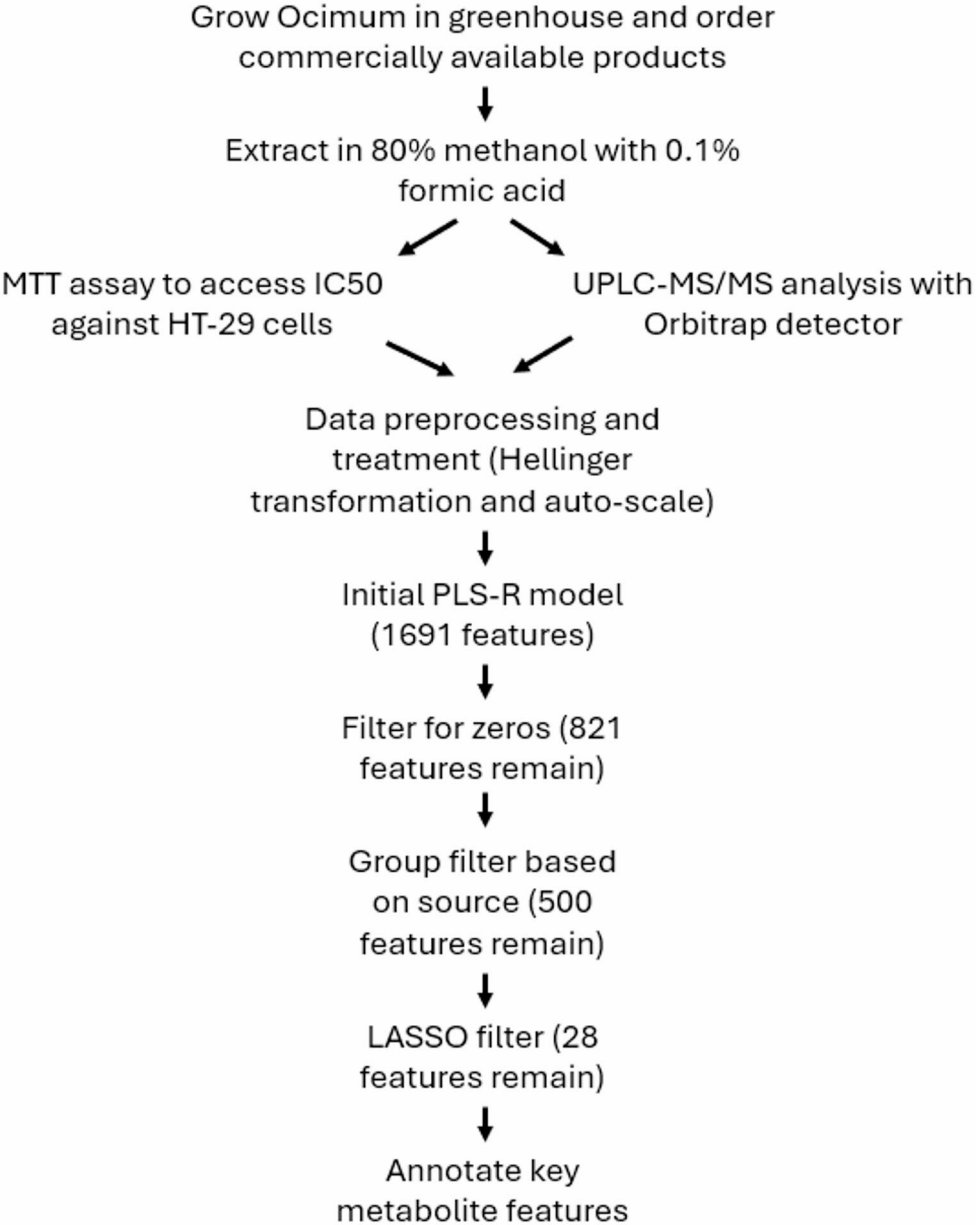
Workflow of the various steps to bioactive compound discovery without fractionation, including sample collection, extraction, testing, processing, and stepwise filtering, leading to annotation of bioactive compounds

For this study, we evaluated the effects of three *Ocimum* species, *O. tenuiflorum*, *O. gratissimum*, and *O. basilicum* against the HT-29 human colorectal cell line. 25 of the 40 materials were grown in a greenhouse at The Pennsylvania State University under controlled conditions, and the remaining materials were ordered online as bulk herbs from various herbal product suppliers. All materials were extracted in 80% methanol in water with 0.1% formic acid prior to MTT cytotoxicity evaluations and untargeted UPLC-MS/MS analysis (Fig. 1).

### Ocimum materials display a range of cytotoxic effects against HT-29 cells

**Fig. 2:**
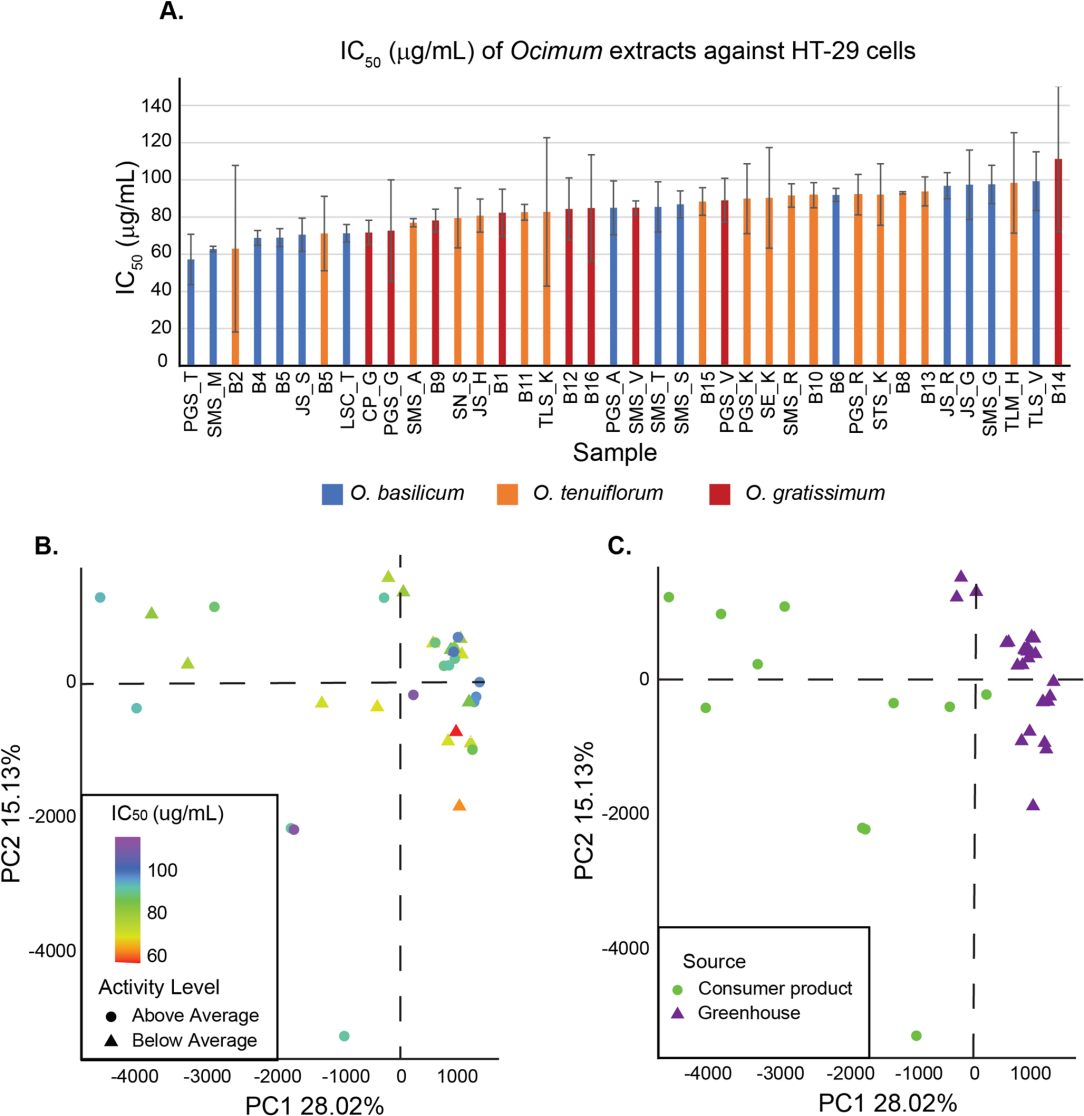
**A** *Ocimum* extracts showed a range of IC_50_ levels, with no evident patterns between species (*O. basilicum* = blue, *O. tenuiflorum* = orange, *O. gratissimum* = red) or source (samples name B# are consumer products). **B** Principal Component Analysis revealed no activity related metabolite patterns. **C** PCA revealed clear differences between material sources (circle = consumer products, triangle = greenhouse grown material)

There was a range of activities amongst the 40 evaluated *Ocimum* materials against human HT-29 colorectal cells (Fig. 2A). We determined the IC_50_ of each extract via an MTT colorimetric assay, which measures cell viability after incubation with a botanical extract or compound of interest. We combined HT-29 cells with extracts of 0–200 µg/ml with 18 replicates of each *Ocimum* material in a 96-well plate. This range of concentrations allowed an accurate calculation of each extract’s IC_50_, which is reported as the average and standard deviation of three separate plating replicates.

According to Nordin et al., extracts with an IC_50_ between 20 µg/ml and 100 µg/ml are considered ‘active’ (but not highly active) in MTT assays (Nordin et al., 2018). All but one of the tested extracts fell within the ‘active’ category (Fig. 2A). The range of activity levels falls within the expected range based on previous *Ocimum* cytotoxicity reports against various cell lines (Dolghi et al., 2021; Harsha et al., 2020; Lam et al. 2017; Torres et al., 2018). We did not find any highly active materials but expected that the observed IC_50_ range of 57.21–116.16 µg/ml would provide a broad enough activity range to identify compounds that explain the observed variation. Since there are no highly active materials, we did not expect to identify any potent cytotoxic compounds. Instead, we aimed to discover medium-active compounds associated with the observed shifts in *Ocimum* biological activity.

The lowest IC_50_ resulted from an *O. basilicum* material; six of the 10 most active samples were *O. basilicum*, and the remaining four were split between *O. gratissimum* and *O. tenuiflorum* (Fig. 2A). This is not to say *O. basilicum* is more active than the other two species; four of the ten least active samples were also *O. basilicum*. Overall, there were no clear species-based patterns guiding *Ocimum* cytotoxicity. Similarly, there is no evidence that greenhouse or consumer products trend toward a higher or lower IC_50_ (Fig. 2A).

We next investigated if there were any underlying patterns in the overall metabolome guiding cytotoxic activity (Fig. 2B). Using the untargeted metabolite profiles, we conducted Principal Component Analysis (PCA); there were not clear patterns between the samples’ metabolite profiles and IC_50_. This indicated that the shifts in bioactivity are likely due to metabolites that are not the main drivers of variation amongst the materials. In addition, the source of the sample, either greenhouse grown or commercial product, did have a clear distinction in the PCA (Fig. 2C), and is a potential driving factor for metabolite diversity; other factors, like environment or processing, could also potentially be the key drivers of metabolite diversity within the basil samples (Abraham et al., 2025).

### Initial feature list is uninformative for predicting biological activity

As unsupervised PCA provided minimal information about the metabolites associated with the differences in *Ocimum* cytotoxicity, we employed a multi-step data filtration and multivariate statistical modeling approach to identify the key metabolites driving bioactivity diversity amongst our materials. We utilized a Partial Least Squares – Regression (PLS-R) model to discover these important features (Wold et al., 2001) based on collinearity and covariance with the dependent variable (bioactivity). The resulting latent components provide insight into the compounds that vary in relation to bioactivity and allow prediction of a new sample’s bioactivity (Wold et al., 2001). Many machine learning models exist with demonstrated relevance to bioactive compound discovery, but PLS-R is by far the most common for botanical investigations and thus we decided to evaluate its potential in this specific workflow (Abraham & Kellogg, 2021).

To evaluate our model’s performance, we split our samples into a test and training set. These sets were balanced to ensure there was an even number of samples above and below the average IC_50_ in each set and that a range of biological activities were represented in model construction and evaluation. Internal validation was conducted via Leave One Out cross validation, and the Root Mean Square Error (RMSE) was used to compare model prediction accuracies. RMSE is a useful validation metric which explains the average variance of the predicted values compared to the expected. Since we log-transformed the IC_50_ values prior to model construction (resulting in values from 4.04 to 4.75, a range of 0.71 on the log scale), the RMSE is reported on the log scale.

The starting dataset contained 1691 features, many of which were likely uninformative to our models. Using this initial dataset resulted in an RMSE of 0.125 for internal validation and 0.160 for external validation (Table 1), meaning the model predictions are within 0.160 units of the actual IC_50_. As the entire IC_50_ range was 0.71, an RMSE of 0.160 is quite high (representing a CV of 22.53%), suggesting a lack of predictive ability in the model due to potentially too many uncorrelated features. Large amounts of uninformative variables are a common hindrance in metabolomic workflows, since many metabolites in a sample are involved in routine cellular activities or are a result of processing and extraction steps (Schiffman et al., 2019). We would like to note that the following steps were performed after our standard workflow in MZmine, and do not include isotope, duplicate, and variance within QC filter samples, and instead focus on the feature list produced after standard processing procedures.

**Table 1:** RMSE of the training set validation (internal validation) and test set validation (external validation) for each PLS-R model. Internal validation was performed using Leave-One-Out cross validation

	No filtering	Blank Filter	Source Filter	LASSO preselection
Training set validation	0.125	0.118	0.011	0.044
Test set validation	0.160	0.158	0.121	0.071

### Data filtration results in an informative feature list

**Fig. 3:**
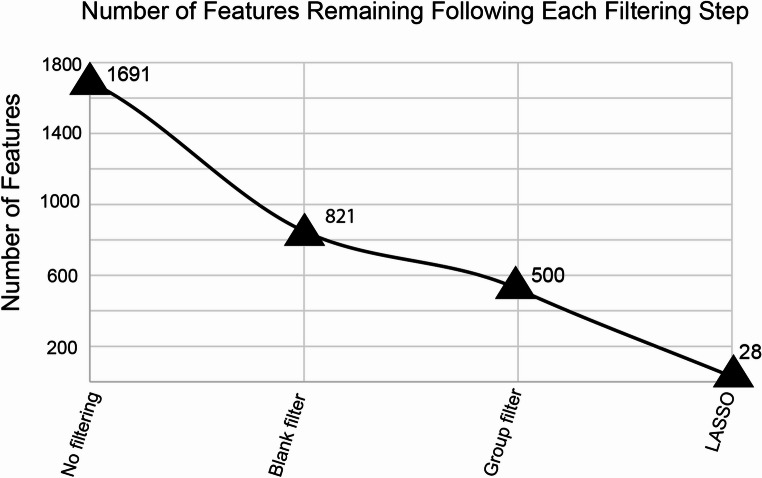
Stepwise data filtration steps reduced the number of features considered in the PLS-R model

To create a more informative feature list, we first applied a 5-fold blank filter. This step is quite common in metabolomics analysis and is implemented in most data processing workflows. Using a concentration based blank filter reduces the likelihood that features included in subsequent modeling are instrument noise or contaminants from sample processing (Ivanisevic & Want, 2019; Schiffman et al., 2019). Since our materials originated from various sources, we grouped them as “greenhouse” or “consumer” products for data filtering to focus our compound search to features present in both material groups, especially since there were no bioactivity trends associated with source. The smaller of these two groups, consumer products, had 16 individuals, so we set a threshold requirement that at least eight samples (or half of the smaller group) have a given feature above the 5-fold value for it to be retained. This reduced the feature list from 1691 to 821 individual metabolites (Fig. 3). Using the blank filtered dataset, our PLS-R model, as described in the methods, resulted in a Root Mean Square Error (RMSE) of 0.118 for internal validation and 0.158 for test set validation. Even though 870 features were removed from the dataset, the prediction accuracy was only slightly improved, indicating that further filtering steps are necessary.

As seen in Fig. 2C, there was a great deal of variability between the greenhouse and consumer product materials. To further restrict our dataset to features relevant to both groups, we continued filtering based on sample source. Our next step was to remove all features that were not present in all 40 samples, or not present in at least half of either the greenhouse or consumer groups. Large amounts of missing values are common in metabolomic datasets, especially considering the expected differences between species and sources. However, we aimed to discover metabolites associated with bioactivity shifts amongst our data and therefore decided to only include more common metabolites. We did not remove all features with missing values since there are likely compounds present in the active, but not inactive materials. Before removing any features, we confirmed that none of the 321 filtered features were correlated with shifts in IC_50_ based on their Pearson’s Correlation Coefficient (data not shown). Implementing this step reduced the feature list by 43% (from 821 to 500, Fig. 3). This filtering step is not common in metabolomic workflows but greatly improved the prediction reliability of our PLS-R model (RMSE = 0.011 for internal validation and 0.121 for test set validation) (Table 1). Other studies use general percent missing value filters to remove any feature with more than 60–80% zeros (Janairo et al., 2021; Schiffman et al., 2019). Our approach was similar; however it was based on a more detailed understanding of our specific data structure.

While these filtering steps greatly improved model predictability, key feature identification is still quite complex when considering 500 compounds. To introduce sparsity, we performed a LASSO regression. LASSO regression models are linear modeling techniques for both feature selection and predictions. This approach introduces a penalty to minimize features that are uninformative in predicting the dependent variable, in this case IC_50_, are removed from the model (Chun & Keleş, 2010). Sparse-PLS (sPLS) is a common computational approach in biochemometrics which combines a LASSO regression to select key variables that are used in a subsequent PLS model (Lê Cao et al., 2011). We constructed our LASSO feature selection tool using Leave One Out cross validation on a training set to select the optimal level of shrinkage to apply to each feature, then performed a second LASSO step to select non-zero values when predicting a test set. Ultimately, the LASSO model selected 28 variables that are informative when predicting IC_50_. While LASSO itself is a predictive model, its RMSE for predicting the test set was 0.137 during this stage, which is not improved over the previous PLS-R models.

Using the 28 features selected from the LASSO regression, we ran a final sPLS-R model to assess the combination of the discussed filtering stages for improving predictive accuracy. Using the 28 features, the PLS-DA model resulted in an RMSE of 0.044 for internal validation and 0.071 for test set validation. This means the model can predict the IC_50_ within 0.071 log units when evaluating new data, much lower than the RMSEs in previous stages. Taken together, this suggests careful data filtration based on an understanding of metadata, like sample source and inherent variation, can improve metabolomics predictive modeling.

### Variable selection

An attractive output of PLS-R models are feature selection plots that easily highlight the metabolites that are most informative for explaining the dependent variable. Two common plots are Selectivity Ratio (SR) plots and Variable Importance in Projection (VIP) plots. The selectivity ratio utilizes the “target projection” variable, which is a univariate representation of the selected number of components which best explain variance in bioactivity. The selectivity ratio is calculated by comparing how much bioactivity variation an individual feature explains to the total variation explained by the target projection variable (the residual variance) (Rajalahti et al., 2009). Features with a higher SR explain more of the total variance in the model and have more power to distinguish the differences in the dependent variable (Rajalahti et al., 2009). SR plots with PLS models have demonstrated success in identifying active compounds from endophytic fungi (Kellogg et al., 2016) and outperformed similar selection methods to discover antioxidants from *Puerariae lobaatae* (Kvalheim, 2020). VIP plots are another common metric for understanding and visualizing the influence a particular feature has on a dependent outcome as well as its influence on model performance and the total variation in the dataset (Wold et al., 2001). Typically, variables with a VIP score above one are considered influential on model performance and should be considered as potential bioactive candidates. Farrés et al. (2015) reported that VIP and SR plots have variable performance based on the type of data and questions, so we considered both approaches in our analysis (Farrés et al., 2015).

**Fig. 4:**
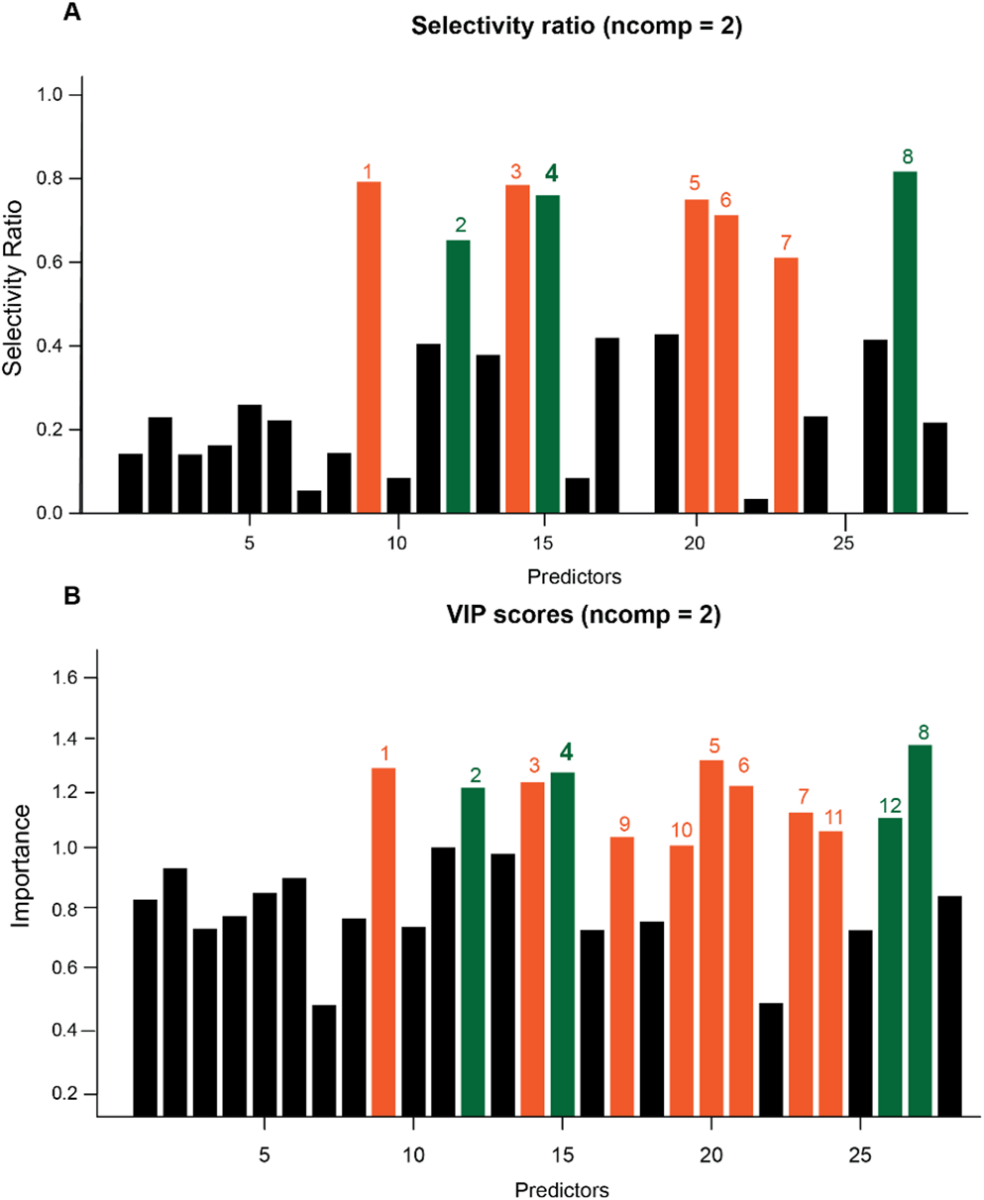
Selectivity ratio (**A**) and Variable Importance in Projection (**B**) plots identify twelve features important for modeling cytotoxicity (IC_50_). Of these twelve, four are associated with increases in activity (green labels, decreasing IC_50_) and eight are associated with decreases in activity (orange labels, increasing IC_50_)

Evaluation of the SR plot resulting from the analysis of 28 features on the first two PLS components, as selected through cross validation (Fig. 4), demonstrates that 8 compounds had a selectivity ratio above 0.5. SR plots can identify compounds associated with positive and negative shifts in activity, and 5 of the 8 compounds were more concentrated in the plants with below average bioactivity (orange numbers) and 3 were related to increased activity (green numbers). The VIP plot for two PLS components identified the same 8 compounds as the SR plot, with the addition of 3 compounds associated with a decrease in bioactivity and 1 associated with an increase in activity.

### Annotation of key features identifies potential bioactive compound

After highlighting the 12 compounds most influential in contributing to shifts in cytotoxicity activity of the basil samples, we pursued annotation via MS2 library matching and molecular networking using the Global Natural Products Social Molecular Networking Site (GNPS). GNPS is an open-source mass spectral repository in which researchers can submit mass spectrums and compound identities. The online tool uses molecular networking to find potential compound annotations based on fragmentation patterns and precursor *m/z* (Nothias et al., 2020). Compound annotation is still complex and time consuming because there are a high number of false positives that result from molecular networking workflows, and manual interpretation of library matches is required (Morehouse et al., 2023).

**Fig. 5:**
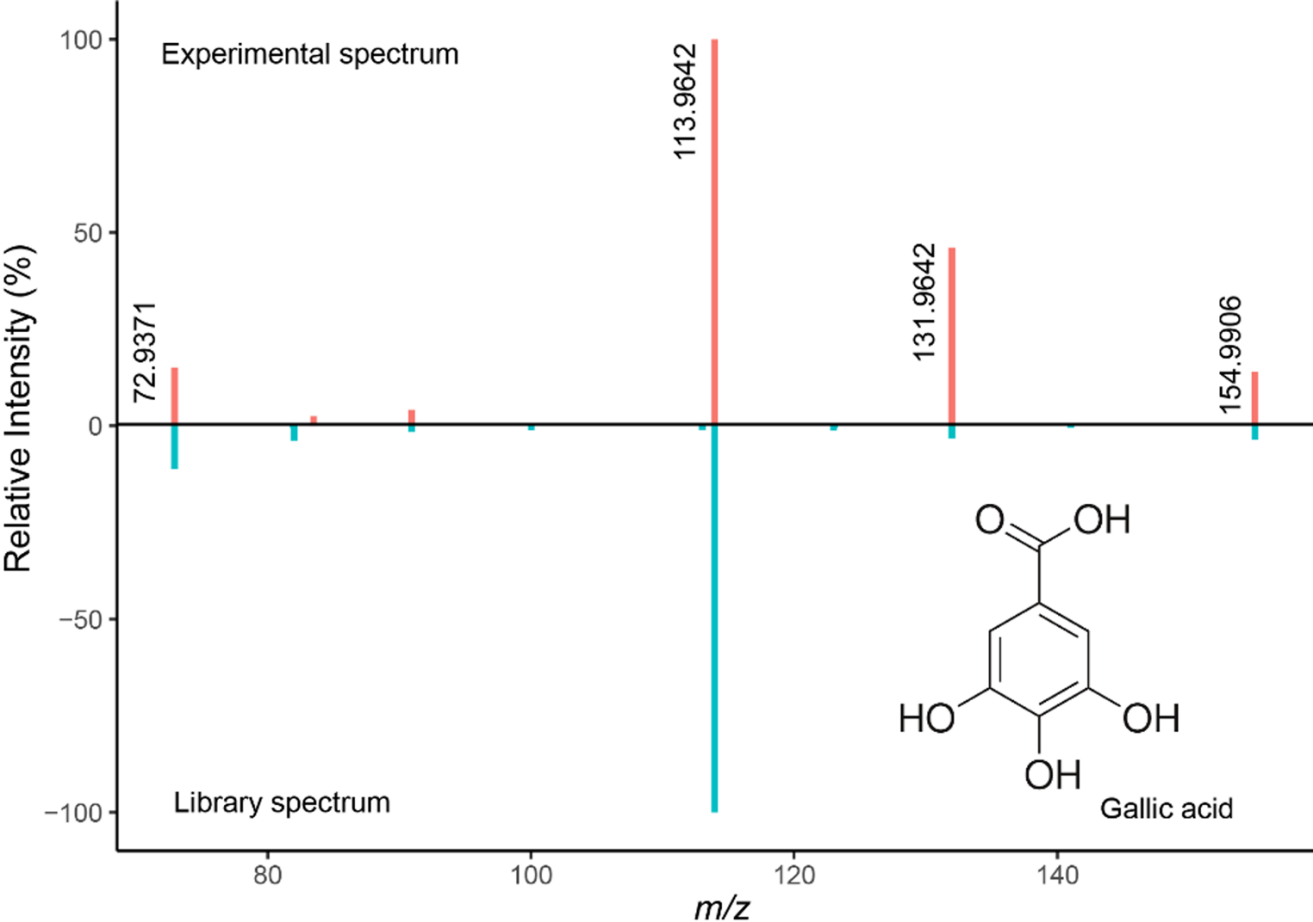
MS2 mirror plot comparing compound **4**’s MS2 spectrum (top, pink) to a library MS2 spectrum for a gallic acid standard (bottom, blue)

One compound returned a potential annotation: compound **4**. We putatively identified this compound as gallic acid based upon retention time comparison with an analytical standard, matching the precursor mass (*m/z* 154.99060), and alignment of major fragmentation peaks at *m/z* 131.9, 113.9, and 72.9 (Fig. 5) (Schymanski et al., 2014). Any discrepancies in the mirror plot with regards to minor peaks are to be expected based on differences in collision energy and method parameters.

**Fig. 6:**
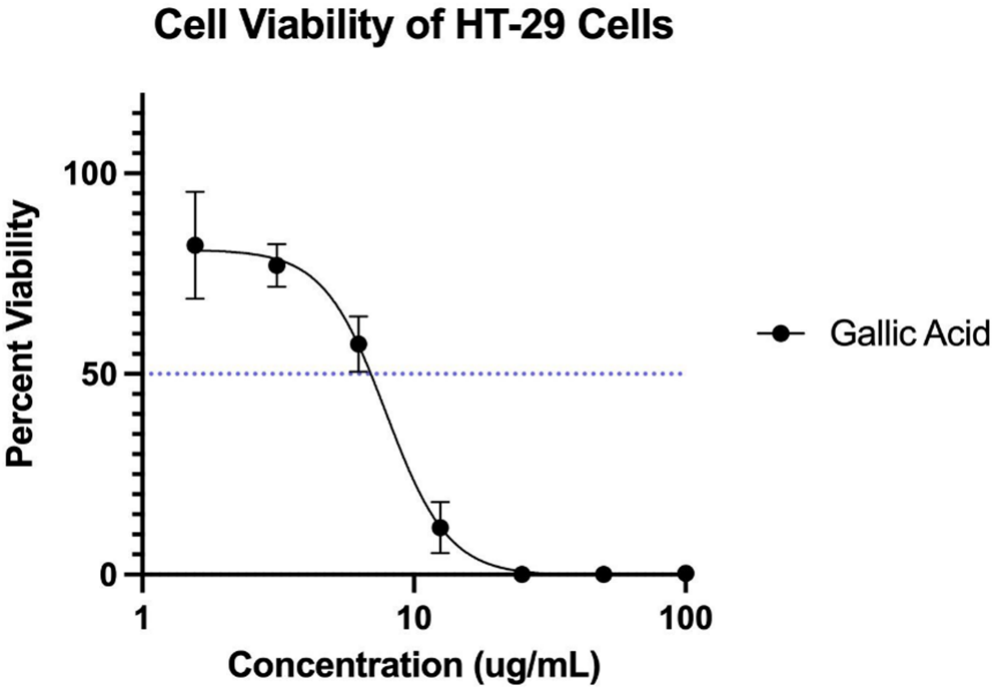
Cytotoxicity assay for gallic acid. Viability of HT-29 cells were assessed using an MTT indicator, and the resulting viability curve is presented. Each dot represents triplicate assays ± SEM

Gallic acid, or 3,4,5-trihydroxynenzoic acid, has been demonstrated to have cytotoxic effects against various cancer cell lines in previous studies. Specific studies have determined that gallic acid has inhibitory activity against HT29 cells, amongst other cell types (Pham et al., 2018). Zhao and Hu (2013) demonstrated that gallic acid decreases HeLa and HTB-35 cancer cell viability, possibly through suppression of ADAM17 and downregulation of various signaling pathways (Zhao & Hu, 2013). To confirm the expected cytotoxic activity, we conducted an MTT assay on HT29 cells with a gallic acid standard (Fig. 6). As expected, based on the previous literature, gallic acid had strong cytotoxic activity against HT29 cells with an IC_50_ of 7.87 ± 0.69 µg/mL. This confirms that the employed filtering and modeling approach successfully identified a bioactive compound associated with cytotoxicity without fractionation.

## Conclusions

The speed of bioactive compound discovery has been hindered by the time and labor requirements of traditional bioassay guided fractionation methods. Using multivariate modeling to integrate untargeted metabolite profiles with bioactivity data, biochemometrics presents an opportunity to improve the rate of key compound identification; however, most biochemometric studies focus on a few fractions of a known active plant to provide gradations of chemical diversity to the model. Aside from time, labor, and material constraints, fractionation of botanical samples may result in the loss of key compounds during processing or separating synergistic metabolites. As an alternative approach, we captured phytochemical variation across a group of taxonomically-related materials with a range of metabolomes and observed bioactivities, instead of fractions of a single active plant, and demonstrated this metabolome-wide association study has the potential to identify bioactive compounds with a single round of assays and extractions. By avoiding fractionation of the parent material, this approach represents a more rapid pathway for bioactive identification, preserves any potential synergistic interactions that could enhance bioactivity, and reduces solvent use (that is required for producing a range of chromatographic fractions).

The chemometric workflow presented in this study is instrumentation-agnostic, being able to be employed with LC-MS, GC-MS, or other high-resolution and high-sensitivity detectors.And while the study did not uncover a novel bioactive compound from basil (which is a widely studied and well-characterized botanical), we confirmed that the novel approach, combining data specific filtering and LASSO preselection prior to Partial Least Squares – Regression analysis, is a potential avenue for discovering active compounds in future studies. It is key to note that this study was undertaken without a priori knowledge of the cytotoxic compounds potentially present in *Ocimum* spp. and was still able to highlight a compound confirmed to possess cytotoxic properties. For other, lesser known botanicals and natural products, when no known compounds are identified from spectral annotation methods, further isolation and chemical characterization would be necessary but could be precisely applied using mass-directed fractionation. As a workflow, future studies will work to determine the level of metabolome similarity between materials required for successful modeling and prediction.

## Supplementary Information

Below is the link to the electronic supplementary material.

Supplementary Material 1—Table S1. Sample code, species/variety information, and extraction yield for *Ocimum* sp. samples used in this study, both grown in the greenhouse or purchased commercially

## Data Availability

Raw spectral data was deposited in the MASSive database (ID: MSV000094012, https://doi.org/10.25345/C5V980317).
